# Supplementary material for: Microplastics’ Impact on the Development of AOM/DSS-Induced Colitis-Associated Colorectal Cancer in Mice
Source: Int J Mol Sci. 2025 Nov 27;26(23):11511. doi: 10.3390/ijms262311511 (PMC12692424; doi:10.3390/ijms262311511)
Supplement: Supplementary file 1 [file ijms-26-11511-s001.zip › Figure S1.pdf]

**Figure S1: Macrophotos of all mice colons**

Colons of 7 mice with AOM/DSS-induced colitis-associated colorectal cancer which **did not receive microplastic (–MP)**

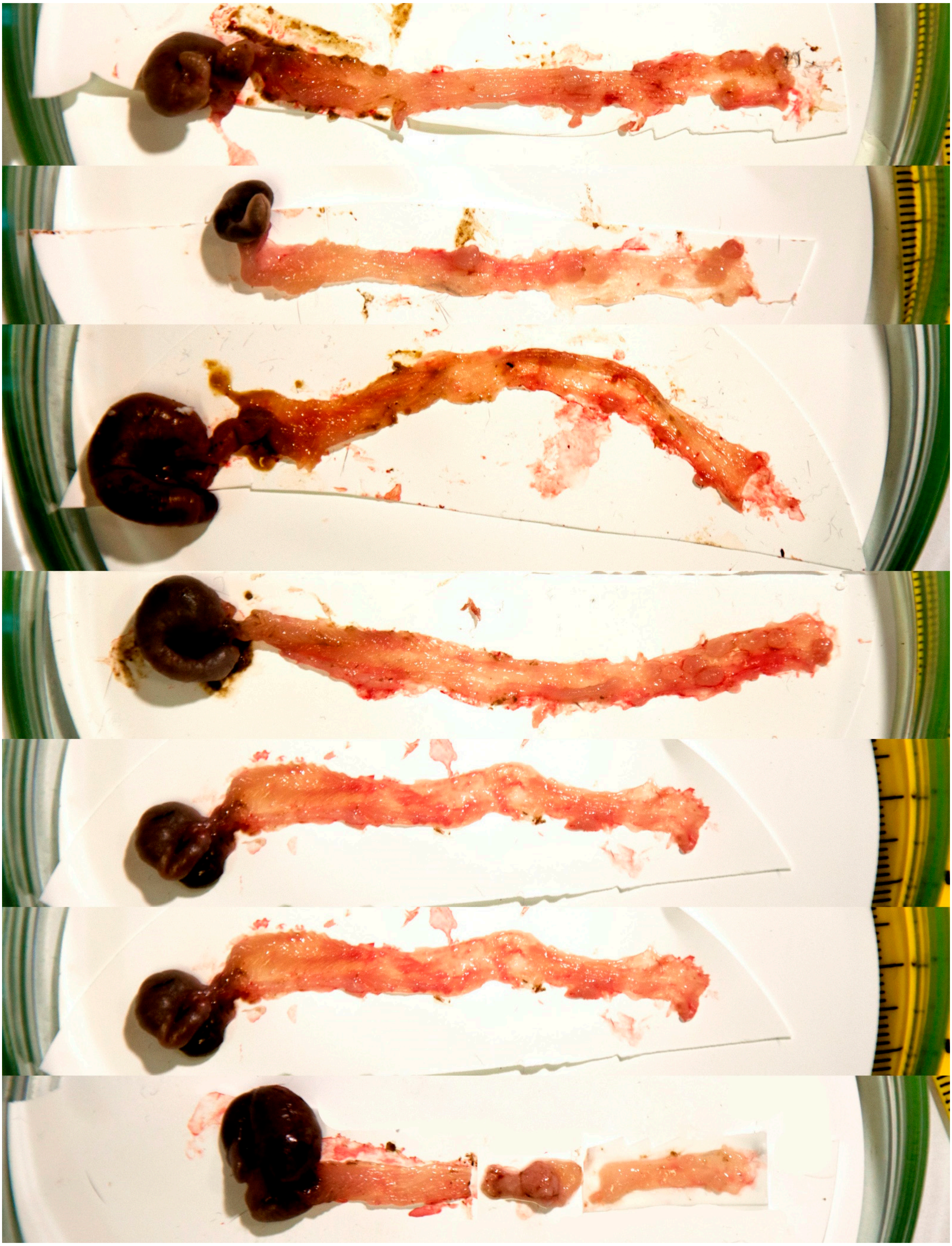

Colons of 7 mice with AOM/DSS-induced colitis-associated colorectal cancer which **received microplastic (+MP)**

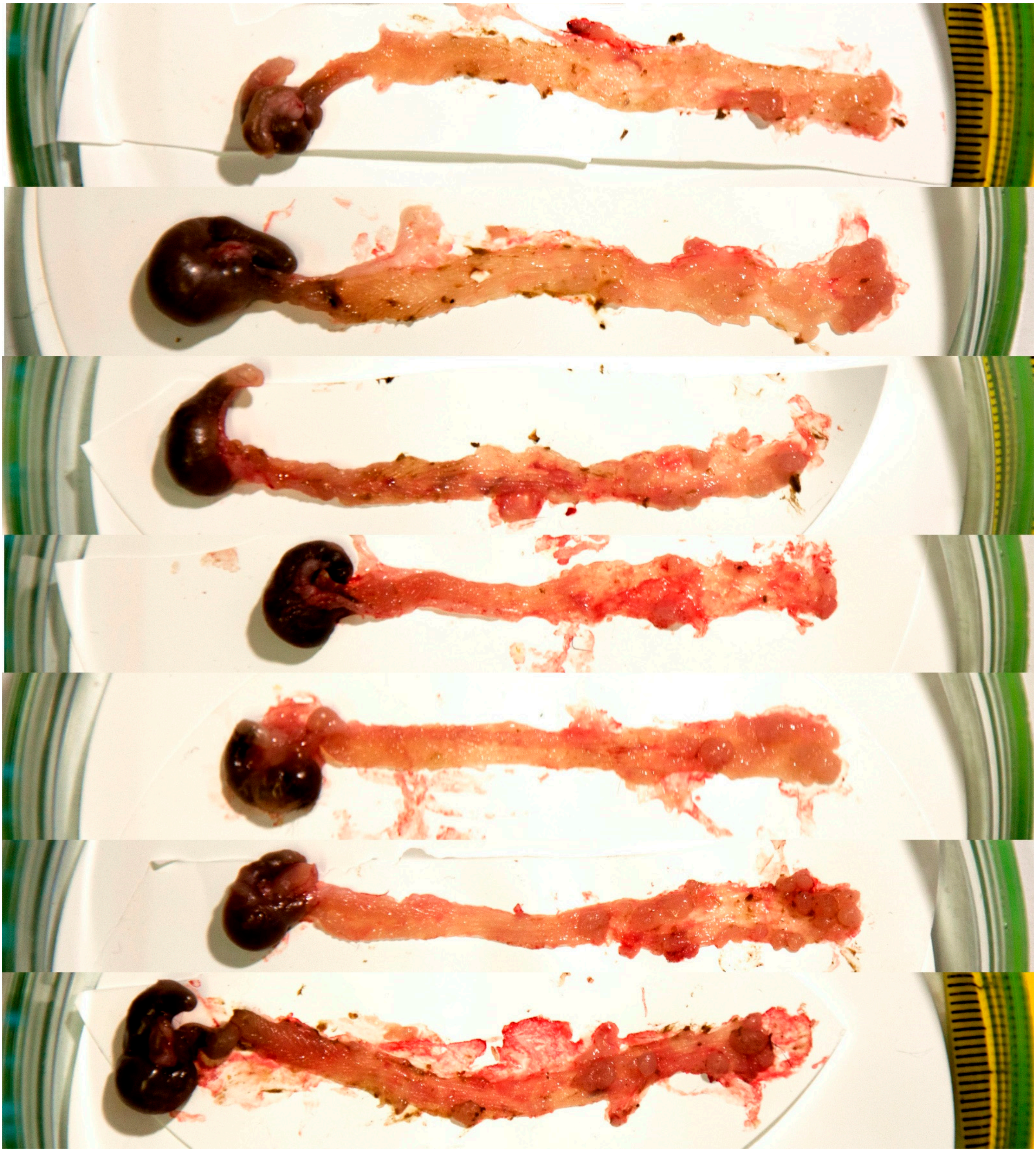

The colon of mouse N7 from group «–MP» was forgotten to be photographed before being cut into sections.
